# Supplementary material for: Anaerobic Gram-Negative Bacteria: Role as a Reservoir of Antibiotic Resistance
Source: Antibiotics (Basel). 2023 May 22;12(5):942. doi: 10.3390/antibiotics12050942 (PMC10215933; doi:10.3390/antibiotics12050942)
Supplement: Supplementary file 1 [file antibiotics-12-00942-s001.zip › antibiotics-2361397-supplementary.pdf]

Supplementary file

Table S1: Minimum inhibitory concentration breakpoints as per the CLSI guidelines

Drugs	Sensitive (S) mg/L	Intermediate (I) mg/L	Resistant (R) mg/L
Metronidazole	≤ 8	16	≥ 32
Clindamycin	≤ 2	4	≥ 8
Imipenem	≤ 4	8	≥ 16
Piperacillin-tazobactam	≤ 16/4	32/4-64/4	≥ 128/4
Chloramphenicol	≤ 8	16	≥ 32
Cefoxitin	≤16	32	≥ 64

Table S2: PCR primers and the thermal cycling parameters used to detect the target AMR genes and IS elements

Gene	Primer sequence (5′–3′)	Size	PCR conditions	Ref.
nim	F-ATGTTCAGAGAAATGCGGCGTAAGCG<br>R-GCTTCCTTGCCTGTCATGTGCTC	458 bp	94°C for 5 min; 35 cycles of 94°C for 30 sec, 63°C for 55 sec, 72°C for 45 sec.	[3]
cfiA	F-ATG GTACCTTCCAACGGG<br>R-CACGATATTGTCGGTCGC	353 bp	94°C for 5 min; 35 cycles of 94°C for 1 min, 52°C for 1 min, 72°C for 1 min.	[54]
IS1186	F-GAGAATCAAGCTTCTCGCC<br>R-CCCCGAATTCGCCTTTGCCCGTA	1-6 kb	94°C for 5 min; 35 cycles of 98°C for 10 sec, 60°C for 30 sec, 68°C for 1 min.	[55,56]
cfiA [IS]	G′-CGCCAAGCTTTGCCTGCCATTAT<br>E′-CTTCGAATTCGGCGAGGGATACATAA	[a]1.6-1.7 kb<br>[b]350 bp	95°C for 1 min; 35 cycles of 95°C for 20 sec, 64°C for 2 min, 72°C for 1 min.	[57]
cepA	F: TTTCTGCTATGTCCTGCCC<br>R: ATCTTTCACGAAGACGGC	780 bp	98°C 10 s, 60°C 30s, 68°C 1 min, 30 cycles	[3]
cfxA	F: ATCGTAGTTTTGAGTATAGCT<br>R: TAAAAGCACTCCGATAACGAT	1010 bp	94°C 1 min, 56.5°C 45 s, 72°C 2 min, 30x	[3]
ermF	F: CGGGTCAGCACTTTACTATTG<br>R: GGACCTACCTCATAGACAAG	466 bp	94°C 30s, 50°C 30 s, 72°C 2 min, 35x	[3]
cat	F: CCTGCCACTCATCGCAGT<br>R: CCACCGTTGATATATCCC	623 bp	94°C 30s, 54°C 30 s, 72°C 2 min, 35x	[58]

F: forward, R: reverse. *cfiA*[IS], *cfiA* gene (350 bp), and the intact segment containing *cfiA* gene and upstream insertion sequence elements (1.6-1.7 kb).

Table S3 (A): PCR reagents and reaction setup for *nim* gene and *cfiA* gene

Reagents	Volume for 25 µl
PCR buffer	0.5 µl
dNTP	1.25 µl
Forward primer	0.5 µl
Reverse primer	0.5 µl
Taq polymerase	0.25 µl
Template DNA	02 µl

$MgCl_2$	01 µl
dH_2O	19 µl
Total	25 µl

Table S3 (B): PCR reagents and reaction setup with master mix for genes (*cfiA*, *ermF*, *cfxA*, *cepA*, *cat*), IS*1186* and *cfiA*[IS]

**Reagents**	**Volume for 20 µl**
Master mix (Sigma REDTaq ReadyMix TM PCR Reaction Mix)	10 µl
Forward primer	0.4 µl
Reverse primer	0.4 µl
Template DNA	02 µl
dH_2O	7.2 µl
Total	20 µl

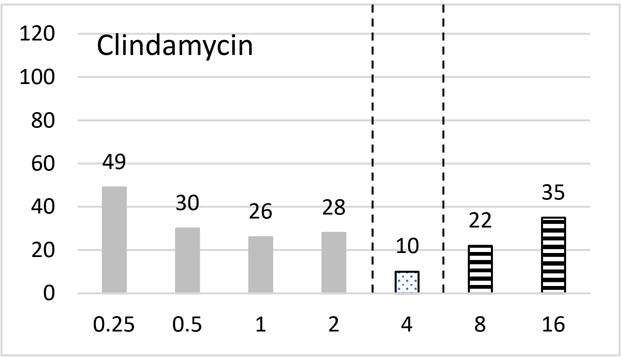

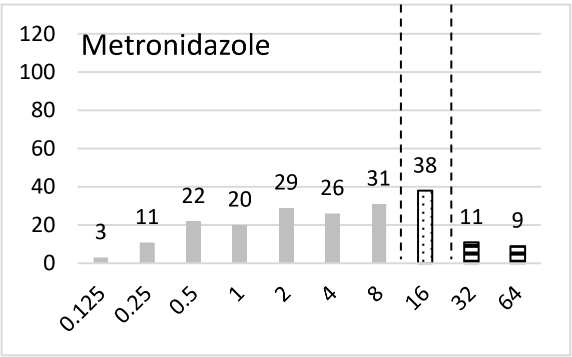

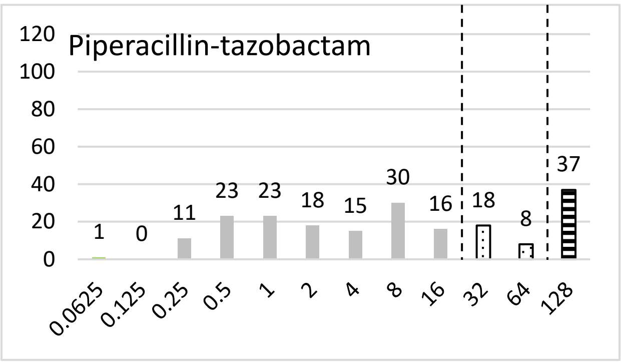

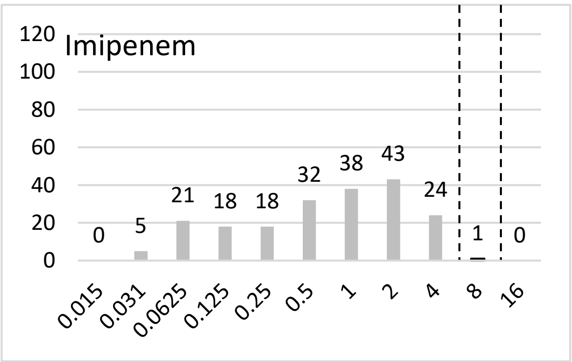

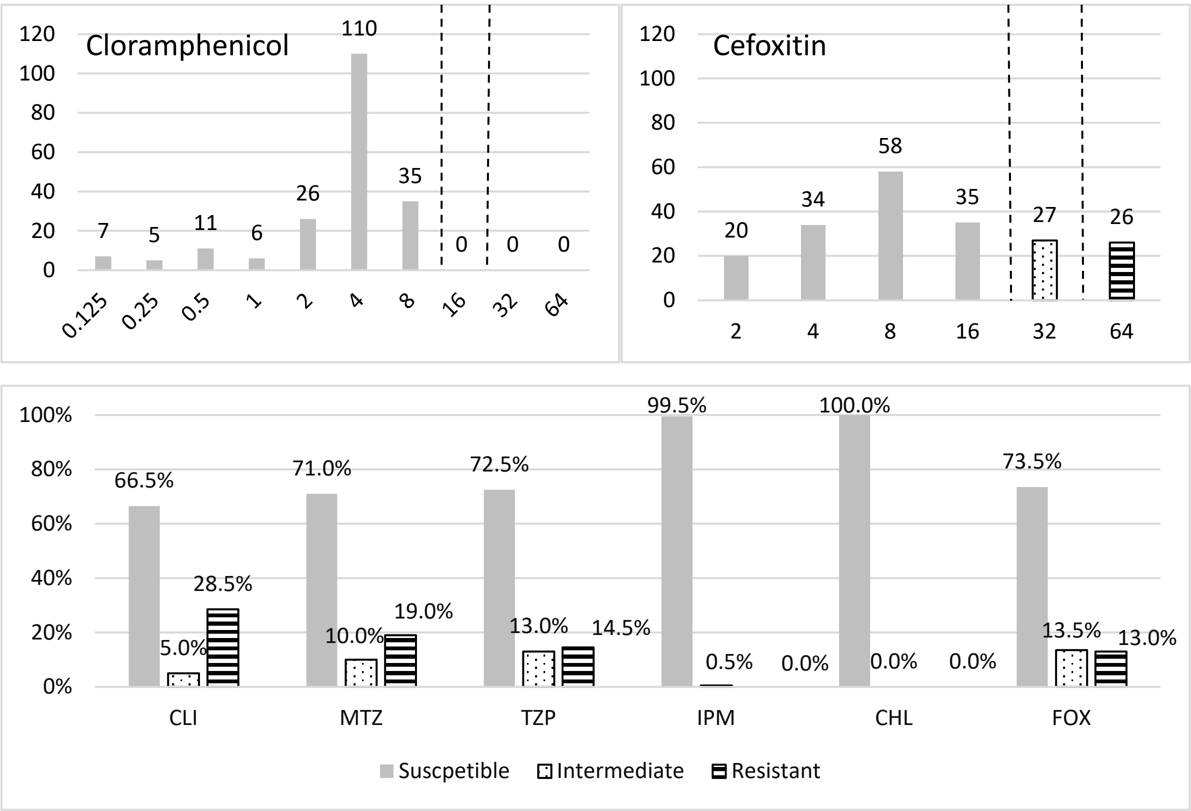

Figure S1: MIC distribution of anaerobic isolates and the overall resistance rate to six antimicrobials. Bars depict the numbers of resistant, intermediate, and susceptible isolates to clindamycin (CLI), metronidazole (MTZ), piperacillin/tazobactam (TZP), imipenem (IPM), chloramphenicol (CHL), cefoxitin (FOX) at different drug concentrations. The broken lines represent clinical breakpoints (mg/L) as per the CLSI guidelines. The x-axis shows drug concentration in (mg/L), and the y-axis shows the number of isolates.

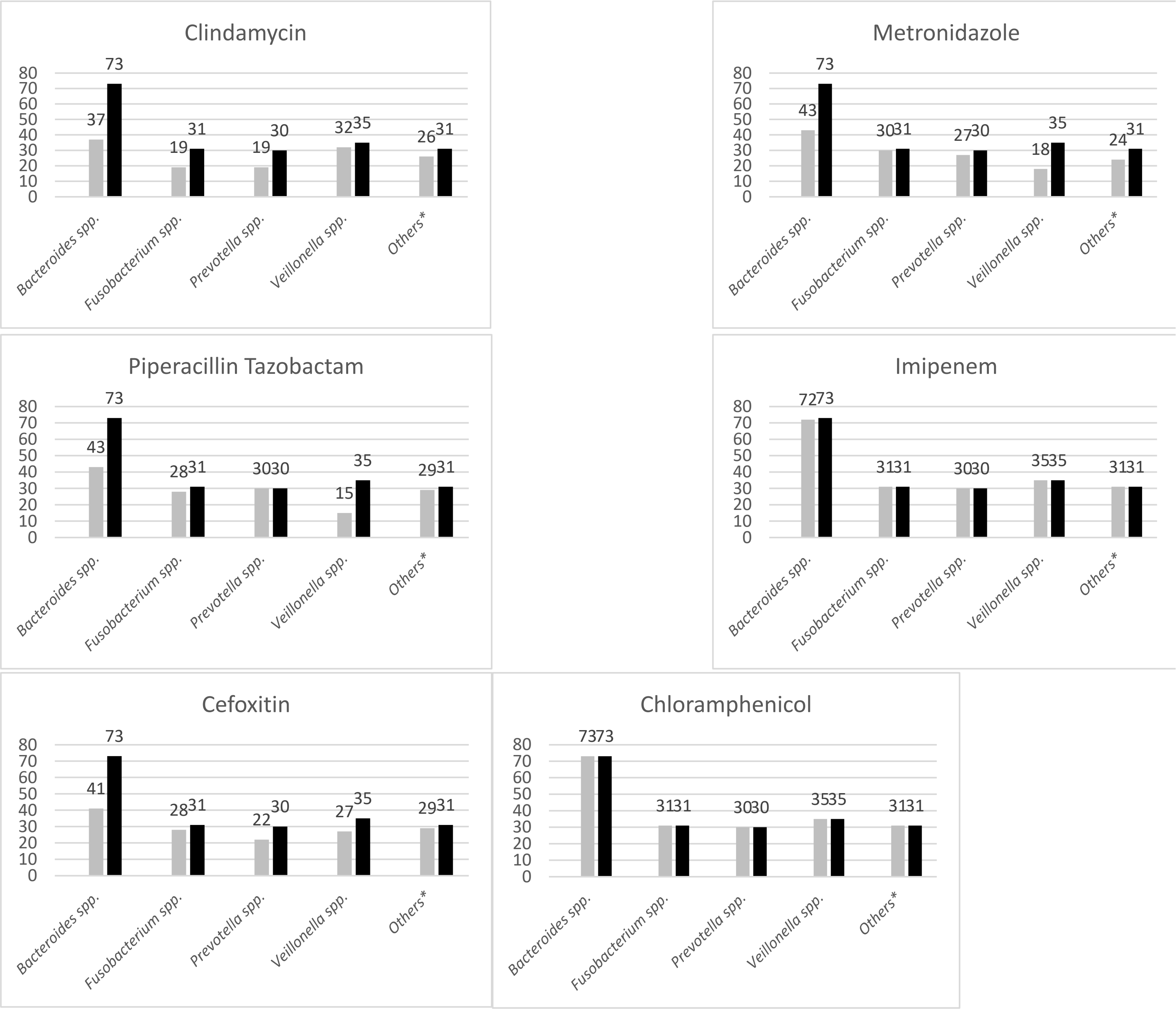

Figure S2: Antimicrobial susceptibility of clinical isolates representing different genera to tested antimicrobials. Others* *Acidaminococcus* spp., *Alistipes* spp., *Anaerobiospirillum* spp., *Bilophilia* spp., *Parabacteroides* spp., *Porphyromonas* spp., *Sutterella* spp. The black and grey bars represent total number of isolates and susceptible isolates, respectively.

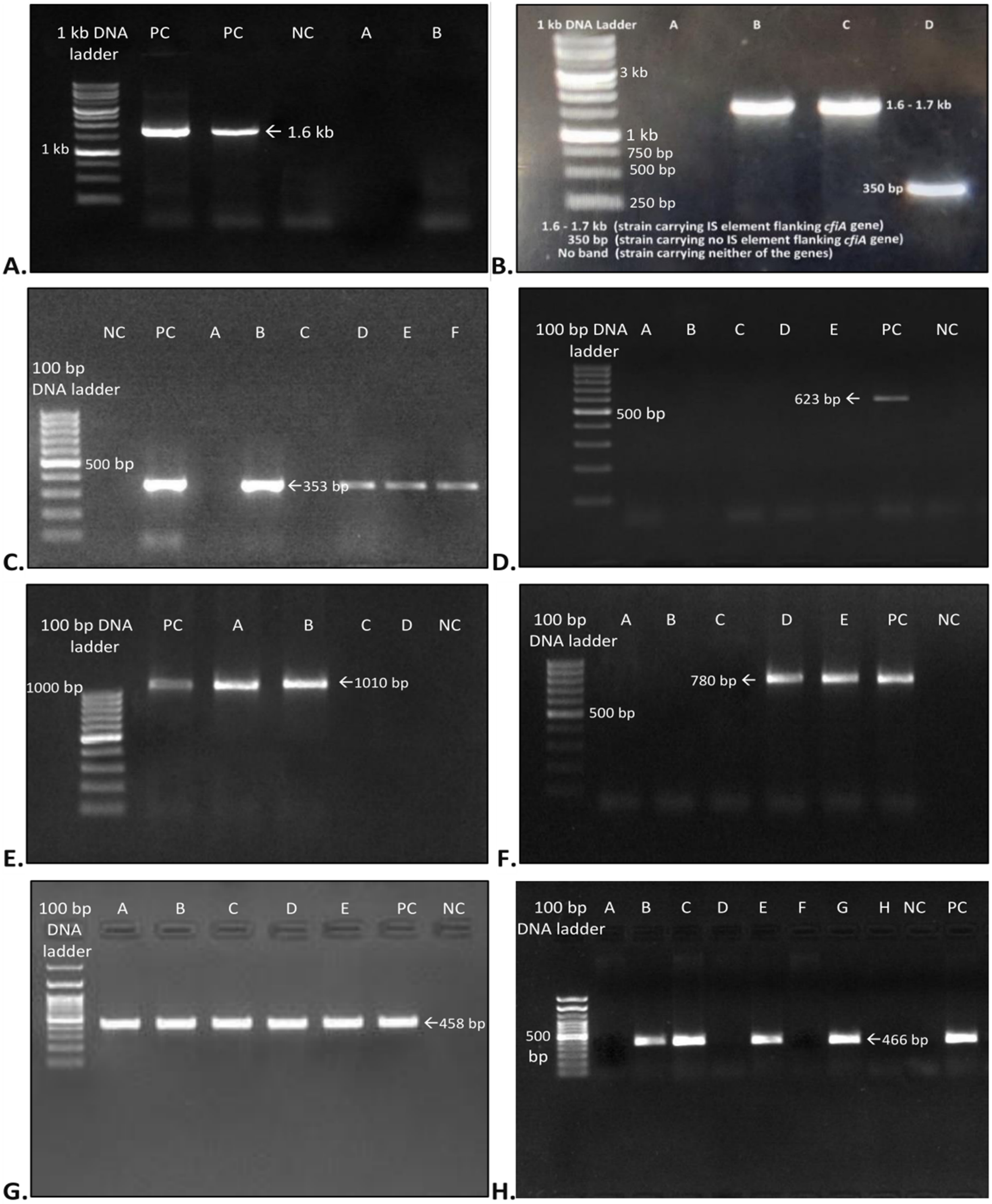

Figure S3: Agarose gel picture for PCR amplified product of A.) *IS*1186 element B.) *cfiA* gene and the flanking IS elements C.) *cfiA* gene D.) *cat* gene E.) *cfxA* gene F.) *cepA* gene G.) *nim* gene H.) *ermF* gene. The first lane contains DNA ladder; PC, positive control; NC, negative control.
